# Eco-efficient recovery of bio-based volatile C2–6 fatty acids

**DOI:** 10.1186/s13068-019-1433-8

**Published:** 2019-04-23

**Authors:** Hee Chul Woo, Young Han Kim

**Affiliations:** Dept. of Chemical Engineering, Pukyong National University, 365 Shinsun-ro, Nam-gu, Busan, 48513 South Korea

**Keywords:** Volatile fatty acid, Fermented product, Extraction, Energy efficient

## Abstract

**Background:**

Volatile fatty acids (VFAs) are produced by fermentation of various bio-sources and human wastes at minimal cost; sometimes, even sources having a prepaid processing fee were used. However, low concentrations of VFAs in water have prevented their commercial production, even with modern separation technologies, due to the high operating costs. We have applied newly developed solvents, selected by chemical structure similarity, to the separation of five different VFAs.

**Results:**

Since most of the water was separated by extraction using hexyl acetate and nonyl acetate, the utilities necessary for solvent recovery and product purification were a fraction of those required by the existing VFAs’ separation processes. The solvents separated almost all the water in the feed at the extraction stage, consuming no energy. The energy use in this study is only 34% of the lowest case use among various processes of either distillation-only or combined extraction–distillation.

**Conclusions:**

The performance evaluation of the proposed VFAs separation process showed that product recovery was 99% and acid purity was 99.5% with eco-scores of 70% lower than those of the current processes.

**Electronic supplementary material:**

The online version of this article (10.1186/s13068-019-1433-8) contains supplementary material, which is available to authorized users.

## Background

Bio-waste, municipal waste, and animal waste are good sources of bioenergy due to their abundance and cost, as well as to their sustainability and environmental benefits. Anaerobic fermentation for biomethane production has been widely used as a practical way for their disposal, and many urban processing plants are currently operated to deliver this methane as heating fuel. When the methane-producing process, methanogenesis, is separately operated from the short-chain-organic-acids producing process, acidogenesis, better control, and efficiency improvements of the whole anaerobic fermentation process are possible [1, 2]. Acidogenesis occurs partially during methanogenesis, unless it is interrupted.

In addition to the above-mentioned benefits of the processes separation, the production of volatile fatty acids (VFAs) increases the products yield and provides wide-application intermediates for producing biofuels by hydrogenation [3], alkanes and alkenes [4], and hydrogen, among others [5]. An improved economics could possibly lead to the direct commercialization of VFAs. Currently, however, their recovery is a cost-ineffective, difficult process due to their low content in the water solution, around 1% and at most 2%.

Various separation techniques commonly used in chemical process industries have been applied for their separation, but no cost-viable process has yet been introduced with detailed process operation results. The currently suggested procedures include extraction [6], adsorption [7], membrane separation [8], pervaporation [9, 10], and reverse osmosis [11]. Because of the low VFAs’ content, the high operating costs of the suggested techniques limit their wide application for VFA recovery. Unless the large amount of water in the feed is efficiently removed, an operating cost reduction is infeasible with any of the existing recovery techniques. However, a solvent of low water solubility, capable of efficiently and selectively dissolving the VFAs, would allow the removal of the large amounts of water present in the feed at low operating cost.

In this work, new solvents were studied for VFAs’ extraction from the fermentation-produced diluted water solution. The process of VFAs extraction, solvent recovery, and product purification was designed for high acid recovery and low-energy demand. The recovery performance, economic evaluation, and lifecycle assessment of the proposed process were studied and compared to other bio-based-products’ recovery processes.

## Methods

### Fermentation procedure

The raw material for VFAs’ recovery was the fermentation product of a diluted alginate solution. Brown alginate (25 g) was dried, ground, and placed in a 500 mL reactor with 225 mL of 3% sulfuric acid solution, and maintained at 120 °C for 250 min [12]. The acid-pretreated solution was neutralized with calcium carbonate and filtered to yield a clear solution. Anaerobic fermentation was carried out for 15.5 days with microorganisms obtained from a municipal wastewater treatment plant in Busan, Korea, under the conditions listed in Additional file 1: Table S1). The VFA composition was determined by gas chromatography (Model GC-17A, Shimadzu, Japan,) using a capillary column (50 m × 0.32 mm × 0.50 µm) and a flame-ionization detector. The VFA composition was not significantly different from other studies regarding VFA fermentation, mostly ˂ 1%. The product composition of the other studies is summarized in Additional file 1: Table S2. The fatty acid production rate depended on the fermentation microorganism. As listed in Additional file 1: Table S2, the fermentation feed also affected VFA production, and the production rates among different fatty acids were largely influenced by the microorganism. High microorganism productivity improved economic feasibility of the VFA recovery process.

### Solvent selection

Although extraction is efficient and, unlike distillation, does not consume much energy, its application is limited to having the proper solvent. Berg categorized extraction solvents in five groups by the strength of their affinity towards water and oxygen for hydrogen bonding [13]. Basically, extraction requires mass transfer in two liquid phases with different distribution of the solute between the phases. Water and organic liquids, mostly water immiscible, provide a heterogeneous liquid that settles into two distinct liquid phases, allowing their easy separation. Finding the proper solvent has been a hurdle for a wider application of this efficient, no-energy-requiring, separation process.

Many experimental results with extraction solvents have been reported, and computer-aided solvent-seeking tools, such as CAMD [14, 15] and COSMO-RS [16], have been developed to utilize the databases compiled with those results. A few studies have focused on how to design ionic-liquid solvents based on the solute molecular structure [17, 18]. For our design, the extraction selectivities of the solvents were determined from their activity coefficients in infinitely dilute solutions, predicted by the non-random two-liquid (NRTL) model using the UNIQUAC Functional-Group Activity Coefficients (UNIFAC) parameters [16]. The UNIFAC parameters are estimated from the functional groups in the molecule [19].

The contribution of the solvent molecular structure to the solute extraction was studied via molecular simulation of the potential forces between solvent and solute [20, 21]. Molecular simulation finds the location of the molecules in a cell, such that the sum of the potentials between solute and solvent atoms is the lowest, namely at equilibrium. When the chemical structures of solute and solvent are similar, their intermolecular potential decreases, resulting in more aggregation. As examples, in a study of protein interactions, the similarity increased the molecular interaction tendency [22], and a molecular simulation result showed that the aggregation of molecules was improved by molecular similarity [23]. In various previous reports, including bio-products separation, the similarity between solutes and solvents has been studied (Table 1) [20, 21, 24, 25].

**Table 1:** Molecular similarity between solvents and solutes in the previous studies

Feed	Solutes	Solvents	References
C9 mixture	1,2,4-Methylbenzene	1,2,4-Chlorobenzene	Cho and Kim [20]
EtBz/*p*-xylene	*p*-Xylene	*p*-Dinitrobenzene	Seo and Kim [21]
Fermented product	Octanoic acid	Methyl stearate in bio-diesel	Kannengiesser et al. [24]
Fermented product	Butyric acid	Hexanoic acid	Rocha et al. [25]
Fermented product	Propionic acid/caproic acid	Hexyl acetate/nonyl acetate	This study

A strong aggregation between structurally similar molecules provides a selection basis for extraction solvents. Esters have been proved to be good solvents for fatty acid extraction [26–28]. Being derived from fatty acids, their molecular similarity supports their selection as solvents for VFAs’ extraction. When the solvent is recovered from the VFA extract, for recycling, it is separated by distillation and some difference in their boiling points facilitates this processing. When the solvent was recovered from the VFA extract for recycling, it was separated by distillation and ˃ 20 °C difference in boiling points was preferred for easy separation. The large amount of water in the extraction feed means that the amount of solvent to be removed is much larger than that of the VFAs, which content is generally less than 2%. High-boiling-point solvents need elevated temperatures at the bottom of the distillation column, requiring high-pressure steam consumption. However, the vapor flow rate in the distillation column is significantly lower than for a low-boiling-point solvent. Especially, the vapor flow in the rectifying section of the column is minimal. The high-boiling solvent consumes less energy and the heat recovered by the cooling of the solvent recycle provides enough heat for preheating the feed to the solvent-recovery column. The recovered heat from the condenser of a low-boiling solvent is at low temperature and, thus, not suitable for use in feed preheating.

**Fig. 1:**
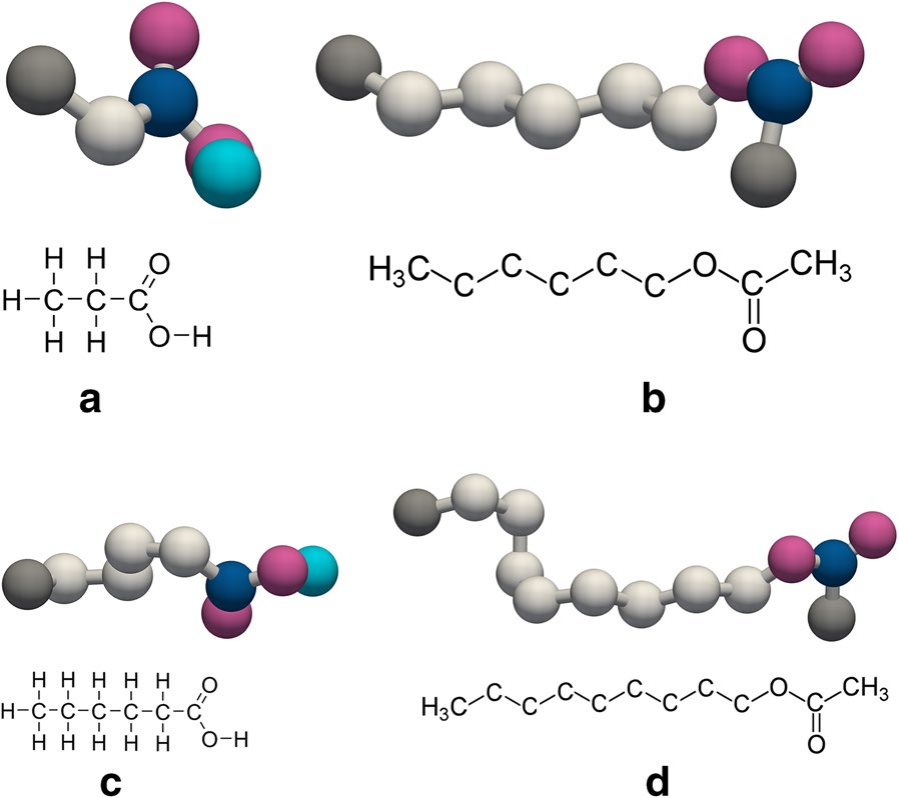
Chemical structures of extract and solvent: **a** propionic acid, **b** hexyl acetate, **c** caproic acid, **d** nonyl acetate. Color code: blue—carbon, grey—methyl group, light grey—methylene group, light blue—hydrogen, and crimson—oxygen

Considering these two aspects, molecular similarity and high-boiling point, two solvents were selected for this study: nonyl acetate for high-carbon-number VFAs and hexyl acetate for low-carbon-number VFAs. A feed containing all fatty acids between acetic acid and caproic acid could not be extracted with a single solvent. Figure 1 shows how the solutes and solvents are similar in their chemical structures, comparing propionic acid to hexyl acetate and caproic acid to nonyl acetate. Although the lengths of the molecular chains are different, the constituent atoms are alike between solvents and solutes.

### Process design

The studied extraction process was a conventional extractive separation: extraction and solvent recovery. Due to the large amount of water in the feed, the extract contained a small amount of water together with the extracted VFAs and the solvent. The solvent was separated from the VFAs and recycled to the extractor. As mentioned before, two solvents were employed, in two identical processes of extraction, solvent recovery and product purification. The commercial process-design software HYSYS (version 7.2) was used in the design [29].

Extractor design is simpler for an atmospheric pressure operation, because only one design parameter is required: number of trays. The number was selected, and equilibrium calculations gave the solution of the material and heat balances. The extraction liquid–liquid equilibrium computations used the NRTL model. Because no vapor phase was present in the equilibrium calculation, the operating pressure of the extraction column did not affect the liquid–liquid equilibrium, which simplified the column design. The binary parameters used in the NRTL model were taken mostly from the built-in HYSYS database: those unavailable in the database were estimated with the UNIFAC group contribution method. The experimental measurements of the liquid–liquid equilibrium in water/acetic acid/hexyl acetate were reported in Ref. [30]. The experimental and computed LLE data using the NRTL model with the UNIFAC-estimated binary parameters are listed in Additional file 1: Table S3. The average absolute error between the experimentally and computationally determined values was 0.023 mol fraction.

**Fig. 2:**
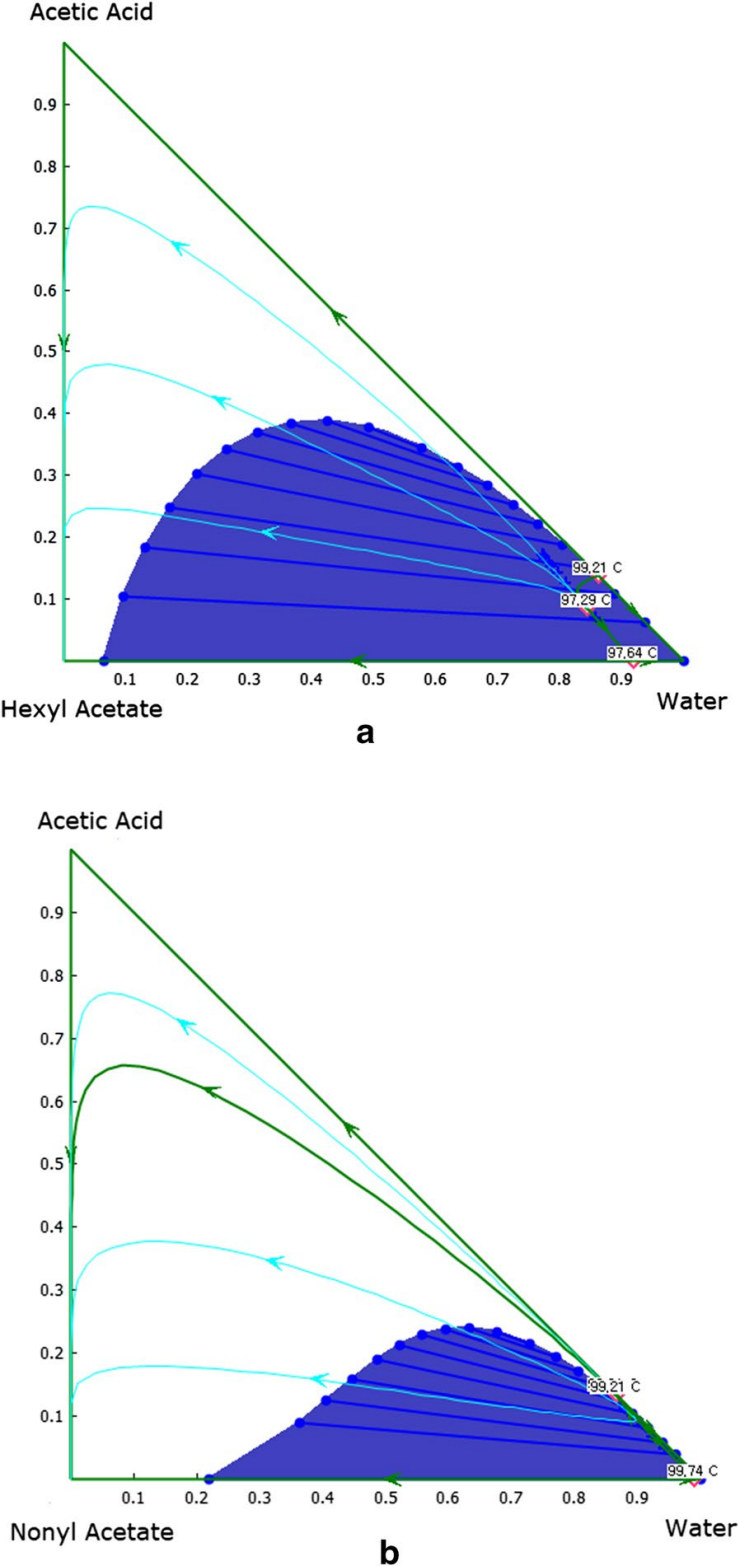
Ternary plot of vapor–liquid–liquid equilibrium: **a** acetic acid/water/hexyl acetate system and **b** acetic acid/water/nonyl acetate system. Green lines in the triangular diagram indicate distillation boundaries, and light blue lines are distillation lines. The numbers are in mole fraction

In contrast, distillation column design requires one more design variable to adjust the product specification. The distillation column design for acetic acid separation had an additional restriction on the column profile: the distribution of the liquid compositions in the column trays could not cross the distillation boundaries, as shown in Fig. 2a, b. The distillation lines are determined by the liquid flow rate in the tray, and the tray profile must follow one of the lines. The separated distillation boundaries and the shape of the distillation lines restrict the free selection of liquid flow and number of trays. The process design was iteratively computed until the products satisfied the desired design targets of VFA recovery and wastewater quality.

The feed VFAs’ composition is summarized in Table 2 and, for the distillation columns design, the VFAs and solvents normal boiling points are also given in the table. Because of the wide distribution of VFAs in the feed, no single solvent extracted all the components at the same time. As explained above, two solvents were used: nonyl acetate for mainly high-carbon-number VFAs and hexyl acetate for acetic and propionic acids. The purification of high-carbon-number VFAs was simple and only one distillation column was needed for the purpose. In contrast, the strong interaction between acetic acid and water required an additional azeotropic distillation process to separate the relatively small amount of water and the solvent recycle. Apart from that difference, the VFAs’ separation processes with the two different solvents were identically designed.

**Table 2:** VFAs’ contents in the fermented product and VFAs and solvents normal boiling points, for reference

Components	Concentration (g/L)	Boiling points (°C)
Acetic acid	5.0862	118.0
Propionic acid	1.9992	141.3
Butyric acid	1.0878	164.1
Valeric acid	1.1368	186.4
Caproic acid	0.49	205.7
Total	9.80	
Hexyl acetate		171.5
Nonyl acetate		224.0

### Techno-economic analysis

Recovery of VFAs is essential in their production from fermented product, because their total content in feed is very low and the recovery strongly affects the production cost of VFAs. The recovery is the ratio of a component amount in product to that in feed. There were separation techniques having different recovery according to carbon numbers in fatty acids. Solvent extraction and membrane separation showed that the higher the carbon number was, the more recovery was yielded [31]. Therefore, a solvent or a system of solvents is necessary to recover at high rates for all fatty acids in feed. Economic feasibility of the proposed process in commercial application was evaluated with utility cost and solvent loss. The previous studies of VFAs’ production indicated that the separation and purification of the VFAs costed 60–80% of production cost [6, 27]. Therefore, a lot of new separation techniques [6–11] have applied to the VFAs processes, but their operating cost was not low enough to be economical in practical processes.

Various combinations of pretreatment and fermentation condition produced a large distribution of VFAs [32, 33]. The biomass pretreatment enhanced the abundance of a certain class of microorganism during fermentation, which resulted in a specific distribution of VFAs [34]. Considering usage and market price of the acids, one can select a proper microorganism and fermentation condition for the most profitable process. The distribution and amount of VFAs were different due to fermentation microorganism, but the proposed process was modified by adjusting solvent amount for different feed. The process was designed for the recovery of all components between C2 and C6 fatty acids.

### Life cycle assessment

The proposed process was composed of extraction and distillation. Environmental impact of the extractor was given only by solvent loss, because no energy was consumed there. On the other hand, environmental impact of the distillation was caused from solvent recovery and product purification by distillation consuming energy. In the proposed process, four eco-scores were calculated using unit scores suggested in the references of distillation [35] and solvent loss [36]. The eco-indicator (EI99) of a material or process indicates its environmental impact based on all emissions and resource uses in terms of damage to human health, ecosystem quality, and resources [37]. The cumulative energy demand (CED) calculates the primary energy demand for the generation of steam in distillation column [36]. The ecological scarcity method (UBP) counts a comparative weighting and aggregate of environmental interventions, calculated from the current pollution and critical pollution possibility [38]. The global warming potential (GWP) was expressed as CO_2_ equivalents [39]. The best scenario in energy duty was based on continuous distillation using waste heat recovery and the worst case on batch distillation using boiler steam [35]. Solvent production using energy of waste-solvent incineration was the best scenario in solvent loss, and the worst was using average European steam generation [36]. The LCA of this study counted heat duty and solvent loss.

## Results and discussion

The process-design results of acid recovery, economics, and environmental sustainability are examined below.

### Process-design results

**Fig. 3:**
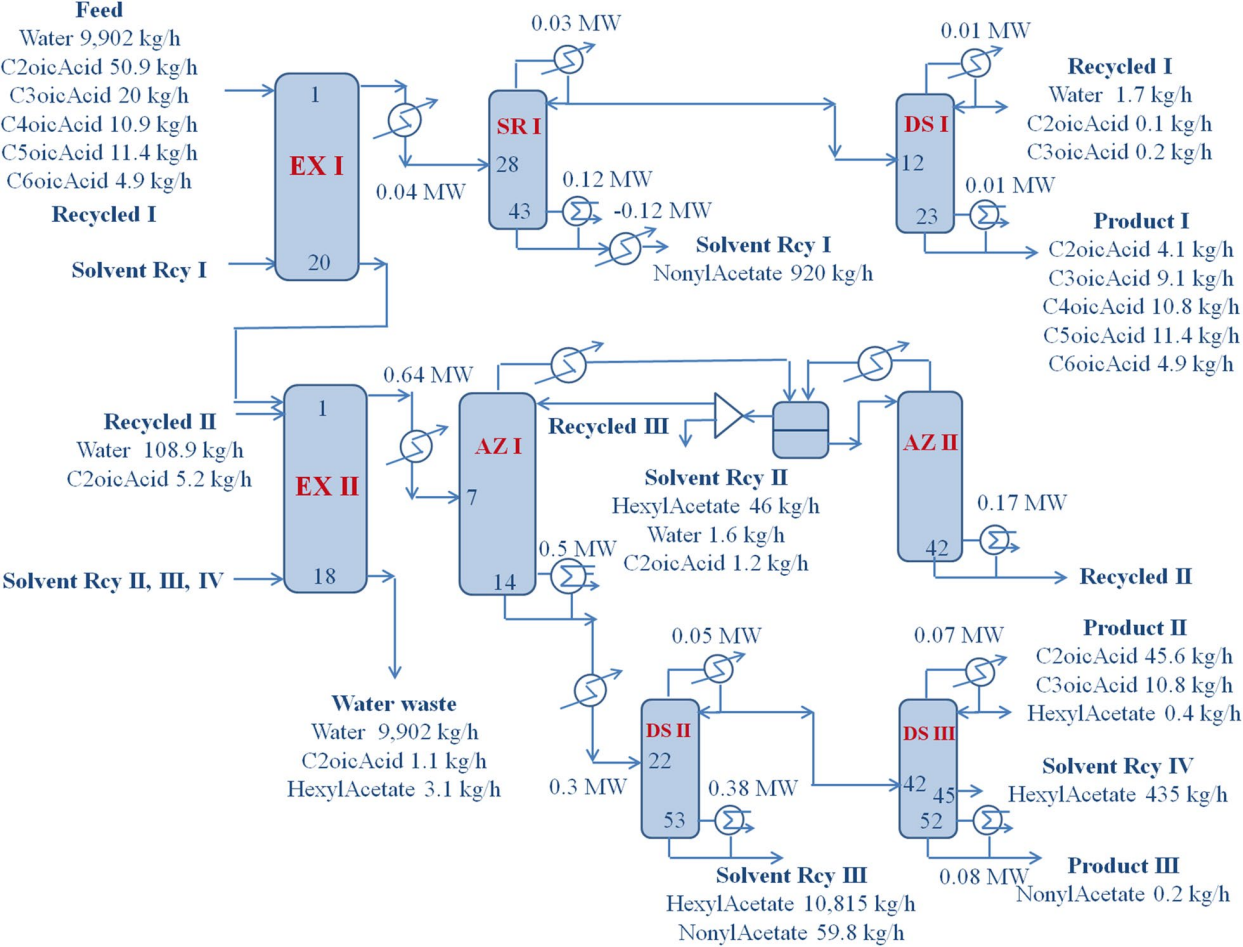
Process flow diagram of the proposed volatile fatty acid recovery process. The numbers in the column indicate tray numbers counted from the top

Because of the large differences in carbon numbers among the fatty acids to be separated, one solvent could not extract all the acids in the feed. Basically, two similar processes of extraction, solvent recovery, and product purification were implemented. However, the second process, separating mostly acetic acid, had a much more complicated purification process due to the high affinity between acetic acid and water, which required more distillation columns, including azeotropic distillation, and consumed more energy. A brief process flow diagram (PFD) describing the two processes of extraction, solvent recovery, and purification is shown in Fig. 3. The numbers in the column indicate tray numbers counted from the top. The first column at top row is an extraction column with top feed inlet and bottom solvent inlet. A summary of the feed and solvent flow rates is shown in the PFD, and detailed flow rates of the constituent components are presented in Additional file 1: Tables S4 and S5. The structural information and operating conditions of the columns are presented in Table 3. All the water in the feed, except for a rate of 1.7 kg/h, was separated in extractor I and fed to the second extractor. The unseparated water was recycled from the distillation column process producing product I to the top of the first extractor. The middle distillation column at the top row of the PFD is a solvent-recovery column that separated the solvent as a bottom product and recycled it to the bottom of the first extractor. The last column separates recycled water and product I.

**Table 3:** Structure and operating conditions of the proposed VFAs’ recovery process

Variable	EX I	SR I	DS I	EX II	AZ I	AZ II	DS II	DS III
Structural								
Tray no.	20	43	23	18	14	42	53	52
Feed	1	28	12	1	7	1	22	42
Solvent	20			18				
Operating								
Pressure (MPa)—top	0.12	0.12	0.12	0.12	0.12	0.12	0.12	0.12
Temp. (°C)								
Overhead	30	123	104	30	114	105	150	127
Bottom	30	234	160	30	178	107	184	232
Feed (kg/h)	10,000	962	42.6	9960	11,531	388	11,368	492
Solv. (kg/h)	920			11,363				
Prod. (kg/h)								
Overhead	962	42.6	2.2	11,531	258	274	492	56.8
Bottom	9960	920	40.3	9906	11,368	114	10,876	435/0.2
Reflux (kg/h)		130	10		95.1	388	7	600
Vap. boil. (kg/h)		1657	53.1		5628	286	4902	1046
Cool. duty (MW)		0.03	0.006				0.05	0.07
Reb. duty (MW)		0.12	0.007		0.50	0.17	0.38	0.08
Pre./recv. (MW)		0.04/0.12			0.64/1.08	–/0.01	0.3	
Comp. (mass frac.)								
Feed								
Acetic acid	0.005	0.004	0.099	0.005	0.005	0.073	0.004	0.093
Product	Ovd.	Ovd.	Bot.	Ovd.	Ovd.	Ovd.	Ovd.	Ovd.
Acetic acid	0.004	0.099	0.101	0.005	0.033	0.084	0.093	0.803

Tray numbers are counted from the top. Equipment names are in the order presented in the PFD

Equipment abbreviation; EX I, extractor I; SR I, solvent recovery I; DS I, distillation column I; EX II, extractor II; AZ I, azeotropic column I; AZ II, azeotropic column II; DS II, distillation column II; DS III, distillation column III

Because nonyl acetate did not extract most of the acetic acid, more than ten times the amount of hexyl acetate was used at the second extractor, at similar stream flows. Because of the high affinity between acetic acid and water it carried out more water when the acids were extracted. The large amount of water removed most of the solvent from the extract and formed two liquid phases. Therefore, the extract distillation had to be an azeotropic separation, as shown by the second and third columns in the middle row of the PFD. The middle decanter, between the columns, separated organic and aqueous phases, after condensing their overhead vapors. All the water products were recycled to the extractor, as in the first section of the process. The bottom product from the first azeotropic column, containing product and solvent, was processed in two distillation columns as at the first section of the process. Because of a small amount of nonyl acetate carried from the first section, the last distillation column has three products, with recycle solvent as a side product. The difficulty of acetic acid solvent extraction was also observed with MTBE; the difficulty was lesser with propionic acid and the least with butyric acid [31, 40].

Solvent contamination of the product is not a problem when it is hydrogenated to yield bio-diesel [40]. Loss of nonyl acetate was not significant, because the small loss (0.3 kg/h) was recovered as product III at a rate of 0.2 kg/h. Loss of hexyl acetate was due to dissolution in the wastewater at a rate of 3.1 kg/h. When the wastewater, which flowed at approximately 10 ton/h, was extracted with cyclohexane at a rate of 250 kg/h, all dissolved hexyl acetate was recovered with the less expensive solvent at an overall loss rate of 5.3 kg/h. Provided that the wastewater containing hexyl acetate is recycled for fermentation in the next batch, the loss would be recovered and no make-up necessary.

The in situ product recovery (ISPR) of VFA has been reported to increase the fermentation capacity and efficiency. The fermentation broth was circulated throughout the hollow-fiber membrane extractor, and the VFAs were recovered with an extractant and oleyl alcohol. The extracted VFAs were stripped with NaOH solution using the other hollow-fiber membrane recovery unit [41–43], and the extracted acid was recovered by distillation [44]. When an appropriate solvent is used in the conventional extractor, complicated extraction equipment, including hollow-fiber membrane extractors, is not necessary. For grain or bio-waste feed, the fermentation broth containing fine solid particles necessitates the use of hollow-fiber membranes, as the particles cause clogging of pores. Because no acute toxicity has been reported for the proposed solvents [45, 46], they were determined to be benign toward the fermentation microorganisms. Their ISPR application is feasible using a common extractor instead of a hollow-fiber membrane extractor, and the recovery and purity of the produced VFAs are higher than those of the current ISPR processes.

### Performance evaluation

The utilization of extraction in the separation process of fermented product significantly reduces downstream processing costs [47, 48], a major hurdle in bio-product utilization [6, 27, 40, 49]. Separation by the direct distillation of the fermented product requires a large amount of energy due to the low VFAs’ content in water, generally 1–2%. The performance of the extraction and distillation process for the bio-product separation is determined by the solvent used in the extraction. The energy demand with the esters used in this study was compared with that of other solvents applied for bio-products and fatty acid separation, as listed in Table 4. The energy use in this study is only 34% of the lowest case use among various processes of either distillation-only or combined extraction–distillation. This significant reduction of energy demand by the proposed separation process is due to the solvents selected for this study. They separated almost all the water in the feed at the extraction stage, consuming no energy, so that only solvent recovery demanded energy. However, the solvent amounts used in our extraction were much lower than those used in other processes with high solvent-to-feed ratios of 5–8 [42]. Another source of energy savings was the high-boiling point of the solvents, which limited the energy-consuming vapor flow in the rectifying section of the solvent-recovery columns. Energy demand in distillation of very low content aqueous solutions depends on the amount of feed, because the thermal properties (heat of vaporization and boiling point) are close to those of water. When comparing separation performance, identical feed and product conditions are necessary for the suggested processes.

**Table 4:** Reboiler heat duty and solvent loss for various bio-products and fatty acid separations

Products	Process	Solvent	Loss (kg/h)	Duty (MW) for ton/h feed	References
Butanol	Distillation			2.71	Aneke and Gorgens [62]
	Extract./distil.	2-Ethyl-1-hexanol	1.6	0.86	
Acetic acid	Distillation			2.59	Li et al. [26].
	Extract./distil.	MTBE	–	0.38	
2,3-Butanediol	Extract./distil.	1-Butanol	0.3	0.43	Haider et al. [63].
VFAs	Extract./distil.	Hexyl acetate Nonyl acetate	0.3 –	0.13	This study

As shown in Additional file 1: Table S4, product recovery was 99% and acid purity was 99.5%. The previous studies found very poor recoveries, especially for low-carbon-number fatty acids [31]. The selected solvents demonstrated near-perfect performance as VFAs’ recovery solvents in terms of product recovery, purity, and energy consumption.

Medium-chain fatty acids were fermented from wastewater, with products including C2–9 fatty acids [43]. These acids were produced by fermentation of acetic acid and ethanol [44]. Household bio-waste was fermented to yield C2–8 fatty acids in total amounts between 8 and 31.5 g/L [24]. The produced fatty acids were extracted using bio-diesel at a recovery of 90 and 95% for heptanoic and octanoic acids, respectively. These extracts were separated by distillation.

Large-scale algae fermentation was conducted with a 300 L reactor at the author’s lab [33]. Various conditions of fermentation resulted in wide ranges of distribution of C2–6 fatty acids, of which a similar condition to this study yielded 3.0 g/L, 1.15 g/L, 0.5 g/L, 0.5 g/L, and 0.3 g/L of acetic, propionic, butyric, valeric, and caproic acids, respectively. Though the VFAs amount was less than this study, their distribution was similar to the feed of the proposed process. As listed in Additional file 1: Table S2, a 100 L reactor with kitchen waste produced another similar distribution of VFAs [32]. When the feed composition is different from this study for the up-scaling process, the amount of solvents is adjusted to maintain VFAs’ recovery with the same arrangement of extractor and distillation columns to the proposed process. There were other separation techniques having different recovery according to carbon numbers in fatty acids. Solvent extraction and membrane separation showed that the higher the carbon number was, the more recovery was yielded [31]. The recovery in the solvent extraction was 61–98% [31]. However, the recovery of this study was nearly complete with all the carbon numbers; 98% for acetic acid, 99% for propionic acid, and 100% for others.

### Economic evaluation

The cost of recovery of VFAs from the fermented VFA feed was determined by considering equipment investment and utility costs. The cost evaluation followed the procedure used in the previous studies [50, 51]. For updating the investment cost, the Marshall & Swift Cost Index, 1593.7 in 2017, was used. The operation was taken as 24 h/300 days per year for the utilities of cost calculation, adjusted for inflation [52].

Table 5 tabulates the investment and utility costs with equipment names separated in two groups, as shown in Fig. 3. Because of the high affinity between water and acetic acid, the second section required more investment cost and more than nine times the utilities costs as compared with the first section, separating high-carbon-number fatty acids. For cost comparison, a similar process for 10,000 ton/year butyric acid production process was used, with investment cost of 8.47 million US dollars, and annual utilities cost of 13.5 million US dollars, at 2014 prices [6]. The costs were only calculated for butyric acid recovery. For investment-cost production-capacity adjustment, an equipment scaling-up exponent of 0.68 [53] was used. The listed costs in Table 5 indicate that our proposed process required 10% less investment and had 62% less utilities cost than the fermented butyric acid process.

**Table 5:** Economics of VFAs’ recovery process

Variable	EX I	SR I	DS I	EX II	AZ I	AZ II	DS II	DS III
Investment								
Column	126.9	53.2	10.9	159.2	42.9	90.7	80.4	72.9
Tray	6.4	1.34	0.2	9.1	1.2	3.5	2.6	2.4
Heat exchanger		55.6	12.0		253.8	99.3	115.9	60.4
Subtotal			266.5					994.3
Total								1261
Utility								
Steam		33.5	2.0		141.7	48.3	108.2	23.3
Water		0.4	0.1		6.5	2.1	0.6	1.0
Subtotal			36.0					331.7
Total								367.7

Units are in thousand U.S. dollars and the utility cost is per annum. Equipment names are in the order presented in the PFD

Equipment abbreviation; EX I, extractor I; SR I, solvent recovery I; DS I, distillation column I; EX II, extractor II; AZ I, azeotropic column I; AZ II, azeotropic column II; DS II, distillation column II; DS III, distillation column III

The proposed process was designed for the recovery of all components between C2 and C6 fatty acids, and it required $0.53/kg fatty acids as utility cost of separation and purification. The utility cost of separation and purification accounts 60% [27] to 80% [6] of whole production cost of acids. Considering mixed acid price of $2.70/kg [54] and solvent loss equivalent to 3% of product amount, the utility cost is low enough to apply the proposed scheme to up-scaling processes.

### Life cycle assessment

Environmental life cycle assessment (LCA) quantifies the environmental impacts of products, services, and organizations [55]. Life cycle assessment was conducted for the proposed VFAs’ recovery process using the life cycle inventory model [35]. Table 6 summarizes the assessment results in four different scores, in the best and worst scenarios. Compared to various bio-products and fatty acid processes, the proposed VFAs’ process gets 70% lower eco-scores. Life cycle assessment was limited to the recovery process operation, but it could be significantly improved when the VFA source became from harvested bio-feed to bio-waste. Further development of VFA recovery processes can help to change discarded bio-waste to eco-friendly resource.

**Table 6:** Environmental eco-scores of various bio-products and VFA processes for 1 kg feed

Products	Scenario	EI-99 [EI-99-pts]	CED [MJ-eq]	UBP’97 [UBP]	GWP [kg CO_2_-eq]	References
Butanol	Best	0.009	2.34	193.5	0.430	Aneke and Gorgens [62]
	Worst	0.033	7.81	230.9	0.560	
Acetic acid	Best	0.004	0.99	83.7	0.189	Li et al. [26]
	Worst	0.014	3.37	98.5	0.263	
2,3-Butanediol	Best	0.005	1.13	95.5	0.215	Haider et al. [63]
	Worst	0.017	3.85	112.9	0.298	
VFAs	Best	0.001	0.35	28.5	0.063	This study
	Worst	0.005	1.15	34.1	0.088	

Eco-scores abbreviation; EI, eco-indicator; CED, cumulative energy demand; UBP, Umweltbelastungspunkte (environmental impact point); GWP, global warming potential

In soybean bio-diesel production, various sustainability indicators were calculated, and their importance of severity in environmental impacts was rated. Oil extraction and purification had fifth significant impact among various processes of the bio-diesel production [56]. Bio-diesel production from algae was analyzed for LCA, and extraction was responsible to 72% of total environmental impact [57]. In the production of bio-diesel derived from fatty oil, the global warming potential (GWP) was 2.04 kg CO_2_-eq/kg oil [58, 59], while that of VFA in the best scenario was 6.5 kg CO_2_-eq/kg VFA. The numbers in Table 6 were of 1 kg feed basis, and 1 ton feed produced 9.66 kg VFAs. Considering low feed content in the fermented product, the difference was expected. The cumulative energy demand (CED) of this study was 36.2 MJ-eq/kg VFA, but that of the bio-diesel was 80.9 MJ-eq/kg oil [58]. Note that bio-diesel production includes biomass cultivation. The eco-indicator 99 in bio-diesel was 0.045 points/kg [60], and that of VFA was 0.103 points. Though the bio-diesel production included various other processes, such as agricultural cultivation, its feed had higher content of raw material than VFAs of fermented product. The eco-score UBP-06 of bio-diesel was 5005 points/kg, in which fatty oil cultivation took around a half [61], and therefore, 2950 points/kg VFA of this study was comparable to the bio-diesel.

## Conclusions

A new VFAs’ separation-from-fermented-product process was proposed for less energy consumption. Two solvents were selected after considering chemical structure similarity, between VFAs and solvents, and boiling points. The proposed extraction, solvent recovery, and product purification processes gave 99% recovery and 99.5% product purity with 10% less investment and 62% reduced utilities cost compared to a similar process of fermented fatty acid production. Life cycle assessment indicated 70% less eco-scores than comparable bio-product processes.

## Additional file

**Additional file 1.** Additional tables.

## Abbreviations

VFA: volatile fatty acid
NRTL: non-random two-liquid
UNIQUAC: Universal Quasichemical
UNIFAC: Universal Quasichemical Functional-Group Activity Coefficients
PFD: process flow diagram
LCA: life cycle assessment
EI99: eco-indicator 99
CED: cumulative energy demand
UBP: ecological scarcity method
GWP: global warming potential
