# Supplementary material for: Eco-efficient recovery of bio-based volatile C2–6 fatty acids
Source: Biotechnol Biofuels. 2019 Apr 23;12:92. doi: 10.1186/s13068-019-1433-8 (PMC6477716; doi:10.1186/s13068-019-1433-8)
Supplement: Supplementary file 1 — Additional file 1. Additional tables. [file 13068_2019_1433_MOESM1_ESM.doc]

**Additional Material**

**“Fermentation Condition, Detailed Stream Flow Rates in Process Flow Diagram, Thermodynamic Model Validation, and VFA Production Comparison”**

# Eco-efficient recovery of bio-based volatile C2-6 fatty acids

# Hee Chul Woo and Young Han Kim^*^

Dept. of Chemical Engineering, Pukyong National University

365 Shinsun-ro, Nam-gu, Busan 48513, South Korea

*Submitted to Biotechnology for Biofuels*

*Correspondence concerning this article should be addressed to:

Dr. Young Han Kim

Address: Department of Chemical Engineering, Pukyong National University, 365 Shinsun-ro, Nam-gu, Busan 48513, South Korea

E-mail: yhkim2@pknu.ac.kr

Tel.: 82-51-629-6436; Fax: 82-51-629-6429

Table S1. Conditions of anaerobic fermentation producing VFAs [12].

| Name | Value |
| --- | --- |
| Working volume (mL) | 200 |
| Temperature (°C) | 35 |
| Nutrients (g/L) | NH_4_HCO_3_ (2), KH_2_PO_4_ (1.0), MgSO_4_·7H_2_O (0.01), NaCl (0.001), Na_2_MoO_4_·2H_2_O (0.001), CaCl_2_·2H_2_O (0.001), MnSO_4_·7H_2_O (0.0015) and FeCl_2_ (0.00278) |
| Concentration ( g-COD/L) | 10 |
| pH adjusting solution | HCl (5N), NaOH (5N) |
| Initial pH | 7.0 |
| Inhibitor | CHI_3_ (50 ppm) |

Table S2. List of fermented volatile fatty acid production from various feeds.

| Feed | Volume (L) | | Acetic | | Propionic | | Butyric | | Valeric | | Caproic | | Total (g/L) | | Ref. | |
| --- | --- | --- | --- | --- | --- | --- | --- | --- | --- | --- | --- | --- | --- | --- | --- | --- |
| Sludge | 14 | | 1.7 | | 0.5 | | 1.05 | |  | |  | | 3.25 | | [64] | |
| Sludge | 0.1 | | 0.96 | | 0.54 | | 0.39 | | 0.11 | |  | | 2.0 | | [65] | |
| Wastewater | 2 | | 2.52 | | 1.73 | | 0.52 | |  | |  | | 4.77 | | [66] | |
| Wastewater | 2.8 | | 0.33 | | 0.23 | | 0.43 | | 0.36 | | 0.13 | | 1.47 | | [67] | |
| Whey waste | 1 | | 1.55 | | 0.56 | | 1.2 | | 0.05 | |  | | 3.36 | | [68] | |
| Microalgae | 6 | | 0.76 | | 0.16 | | 2.97 | | 0.2 | | 0.13 | | 4.22 | | [69] | |
| Kitchen waste | 100 | | 7.3 | | 5.2 | | 1.4 | | 2.0 | |  | | 15.9 | | [32] | |
|  | |  | |  | |  | |  | |  | |  | |  | |  |

References

64. Liu HB, Wang YY, Yin B, Zhu YF, Fu B, Liu H. Improving volatile fatty acid yield from sludge anaerobic fermentation through self-forming dynamic membrane separation. Bioresource Technology 2016;218:92-100.

65. Yang L, Chen ZL, Yang JF, Liu Y, Wang J, Yu YJ, Gao XM. Removal of volatile fatty acid in landfill leachate by the microwave-hydrothermal method. Desalination and Water Treatment 2014;52:4423-9.

66. Horiuchi JI, Shimizu T, Tada K, Kanno T, Kobayashi M. Selective production of organic acids in anaerobic acid reactor by pH control. Bioresource Technology 2002;82:209-13.

67. Yu HQ, Fang HHP. Acidogenesis of gelatin-rich wastewater in an upflow anaerobic reactor: influence of pH and temperature. Water Research 2003;37:55-66.

68. Bengtsson S, Hallquist J, Werker A, Welander T. Acidogenic fermentation of industrial wastewaters: Effects of chemostat retention time and pH on volatile fatty acids production. Biochemical Engineering Journal 2008;40:492-9.

69. Gruhn M, Frigon JC, Guiot SR. Acidogenic fermentation of Scenedesmus sp.-AMDD: Comparison of volatile fatty acids yields between mesophilic and thermophilic conditions. Bioresource Technology 2016;200:624-30.

Table S3. Experimental and computed mole fractions in liquid-liquid equilibrium of water/acetic acid/hexyl acetate system. Component abbreviations; WA: water, AA: acetic acid

|  | Experimental [30] | | | | | | | | Estimated | | | | | | |
| --- | --- | --- | --- | --- | --- | --- | --- | --- | --- | --- | --- | --- | --- | --- | --- |
|  | Aqueous | | | Organic | | | | | Aqueous | | | | Organic | | |
| Temperature | WA | AA | | WA | | | AA | | WA | | AA | | WA | | AA |
| 283.16 | 0.9436 | 0.0562 | | 0.0732 | | | 0.0732 | | 0.9404 | | 0.0590 | | 0.0549 | | 0.0706 |
|  | 0.8989 | 0.1005 | | 0.1133 | | | 0.1371 | | 0.8920 | | 0.1062 | | 0.0696 | | 0.1330 |
|  | 0.8473 | 0.1513 | | 0.1542 | | | 0.2032 | | 0.8397 | | 0.1557 | | 0.0914 | | 0.2033 |
|  | 0.7966 | 0.1993 | | 0.2086 | | | 0.2646 | | 0.7883 | | 0.2024 | | 0.1206 | | 0.2715 |
|  | 0.7317 | 0.2578 | | 0.2835 | | | 0.3216 | | 0.7266 | | 0.2548 | | 0.1671 | | 0.3439 |
| 298.14 | 0.9448 | 0.0550 | | 0.0831 | | | 0.0723 | | 0.9437 | | 0.0557 | | 0.0602 | | 0.0721 |
|  | 0.8914 | 0.1080 | | 0.1319 | | | 0.1498 | | 0.8873 | | 0.1107 | | 0.0808 | | 0.1498 |
|  | 0.8384 | 0.1592 | | 0.1840 | | | 0.2202 | | 0.8337 | | 0.1612 | | 0.1083 | | 0.2253 |
|  | 0.7710 | 0.2224 | | 0.2402 | | | 0.2781 | | 0.7769 | | 0.2121 | | 0.1481 | | 0.3006 |
| 318.15 | 0.9989 | 0.0011 | | 0.0547 | | | 0.0024 | | 0.9984 | | 0.0015 | | 0.0515 | | 0.0021 |
|  | 0.9446 | 0.0552 | | 0.1009 | | | 0.0742 | | 0.9455 | | 0.0540 | | 0.0679 | | 0.0762 |
|  | 0.8819 | 0.1171 | | 0.1585 | | | 0.1601 | | 0.8837 | | 0.1141 | | 0.0955 | | 0.1672 |
|  | 0.8420 | 0.1556 | | 0.1882 | | | 0.2025 | | 0.8493 | | 0.1466 | | 0.1162 | | 0.2178 |
|  | 0.7821 | 0.2112 | | 0.2431 | | | 0.2628 | | 0.7968 | | 0.1944 | | 0.1563 | | 0.2904 |
|  | 0.7174 | 0.2647 | | 0.3175 | | | 0.3134 | | 0.7407 | | 0.2422 | | 0.2102 | | 0.3536 |
|  |  | |  | |  |  | |  | |  | |  | |  | |

Table S4. Component flow rates of feed, product, and recycle streams in the VFAs recovery process. Units are kg/h. Stream name abbreviations; PR1: Product I, PR2: Product II, PR3: Product III, WA: Waste water, RC1: Recycle I, RC2: Recycle II, RC3: Recycle III. Stream names are shown in the PFD.

| Components | Feed | | PR1 | | PR2 | | | | PR3 | | WA | | RC1 | | RC2 | | RC3 | |
| --- | --- | --- | --- | --- | --- | --- | --- | --- | --- | --- | --- | --- | --- | --- | --- | --- | --- | --- |
| Water | 9,902 | | 0.0 | | 0.0 | | | | 0.0 | | 9,902 | | 1.73 | | 108.9 | | 3.12 | |
| Acetic acid | 50.86 | | 4.09 | | 45.61 | | | | 0.0 | | 1.12 | | 0.15 | | 5.15 | | 2.23 | |
| Propionic acid | 19.99 | | 9.06 | | 10.78 | | | | 0.0 | | 0.10 | | 0.23 | | 0.0 | | 0.86 | |
| Butyric acid | 10.88 | | 10.82 | | 0.02 | | | | 0.0 | | 0.01 | | 0.13 | | 0.0 | | 0.0 | |
| Valeric acid | 11.37 | | 11.37 | | 0.0 | | | | 0.0 | | 0.0 | | 0.0 | | 0.0 | | 0.0 | |
| Capric acid | 4.90 | | 4.90 | | 0.0 | | | | 0.0 | | 0.0 | | 0.0 | | 0.0 | | 0.0 | |
| Total VFAs | 98.0 | | 40.23 | | 56.41 | | | | 0.0 | | 1.23 | |  | |  | |  | |
|  |  | |  | |  | | | |  | |  | |  | |  | |  | |
| Hexyl acetate |  | | 0.0 | | 0.39 | | | | 0.01 | | 3.06 | | 0.0 | | 0.0 | | 88.86 | |
| Nonyl acetate |  | | 0.11 | | 0.0 | | | | 0.19 | | 0.0 | | 0.0 | | 0.0 | | 0.0 | |
|  |  | | |  | |  | |  | |  | |  | |  | |  | | |
|  | |  | |  | | |  |  | |  | |  | |  | |  | |  |

Table S5. Component flow rates of solvent recoveries, intermediate raffinate, heavy liquid in decanter, and make-up streams in the VFAs recovery process. Units are kg/h. Stream name abbreviation; SR1: Solvent Recovery I, SR2: Solvent Recovery II, SR3: Solvent Recovery III, SR4: Solvent Recovery IV, RF1: Raffinate from extractor I, HD: Heavy decant, MK1: Make-up to SR1, MK2: Make-up to SR2. Stream names are shown in the PFD.

| Components | SR1 | | | SR2 | | SR3 | | SR4 | | RF1 | | | HD | MK1 | MK2 |
| --- | --- | --- | --- | --- | --- | --- | --- | --- | --- | --- | --- | --- | --- | --- | --- |
| Water | 0.0 | | | 1.63 | | 0.0 | | 0.0 | | 9902 | | | 353.4 |  |  |
| Acetic acid | 0.0 | | | 1.17 | | 0.0 | | 0.03 | | 46.77 | | | 28.11 |  |  |
| Propionic acid | 0.0 | | | 0.45 | | 0.08 | | 0.08 | | 10.93 | | | 2.23 |  |  |
| Butyric acid | 0.0 | | | 0.0 | | 0.16 | | 0.0 | | 0.06 | | | 0.0 |  |  |
| Caleric acid | 0.0 | | | 0.0 | | 0.0 | | 0.0 | | 0.0 | | | 0.0 |  |  |
| Capric acid | 0.01 | | | 0.0 | | 0.0 | | 0.0 | | 0.0 | | | 0.0 |  |  |
|  |  | | |  | |  | |  | |  | | |  |  |  |
| Hexyl acetate | 0.0 | | | 46.0 | | 10,815 | | 435 | | 0.0 | | | 3.71 | 0.0 | 3.42 |
| Nonyl acetate | 920.04 | | | 0.0 | | 59.8 | | 0.22 | | 0.19 | | | 0.0 | 0.30 | 0.0 |
|  |  |  |  | |  | |  | |  | |  |  | | | |
